# *Lactobacillus rhamnosus* GG ATCC53103 and *Lactobacillus plantarum* JL01 improved nitrogen metabolism in weaned piglets by regulating the intestinal flora structure and portal vein metabolites

**DOI:** 10.3389/fmicb.2023.1200594

**Published:** 2023-06-29

**Authors:** Feng He, Xueying Jin, Chunfeng Wang, Jingtao Hu, Shuai Su, Lei Zhao, Tingting Geng, Yuan Zhao, Li Pan, Nan Bao, Hui Sun

**Affiliations:** ^1^College of Animal Science and Technology, Jilin Agricultural University, Changchun, China; ^2^Ministry of Education Laboratory of Animal Production and Quality Security, Jilin Agricultural University, Changchun, China; ^3^Jilin Provincial Key Laboratory of Animal Nutrition and Feed Science, Jilin Agricultural University, Changchun, China; ^4^College of Animal Science and Veterinary Medicine, Heilongjiang Bayi Agricultural University, Daqing, Heilongjiang, China

**Keywords:** metabolites, nitrogen metabolism, weaned piglets, *Lactobacillus rhamnosus* GG ATCC53103, *Lactobacillus plantarum* JL01

## Abstract

At present, most studies have shown that probiotics have a positive regulatory effect on the nutritional metabolism of the body, but the mechanism is still unclear. Here, 48 piglets were divided into four groups. The control group was not fed probiotics, the Lac group was fed *L. Rhamnosus* GG ATCC53103, the Rha group was fed *L. Plantarum* JL01, and the mix group was fed two types of probiotics. Nitrogen metabolism and mRNA levels of mTOR and S6K in skeletal muscle were observed in each group. Then, metagenome and non-targeted metabonomics were used to observe the changes of intestinal microorganisms and plasma metabolites in portal channels after probiotics feeding. Finally, we combined the results of omics analysis to reveal the mechanism of probiotics on nitrogen metabolism in weaned piglets. The results showed that *L. Rhmnosus* GG ATCC53103 and *L. Plantarum* JL01 increased nitrogen apparent digestibility, nitrogen deposition rate, and nitrogen utilization rate of weaned piglets (*P* < 0.05); the relative expression of mTOR and SK6 mRNA in skeletal muscle increased significantly (*P* < 0.05). When *L. rhamnosus* GG ATCC53103 and *L. plantarum* JL01 were combined, we found that *Clostridium* and *Prevotella* significantly increased in the jejunum (*P* < 0.05). The relative abundance of *Lactobacillus, Ruminococcus, Streptococcus*, and *Prevotella* in the ileum increased significantly (*P* < 0.05). Compared with the control group, L-Tryptophan, 3-Phosphonyloxypyruvate, cis-Aconitate, and Carbamoyl phosphate were significantly increased in the mixed group portal vein. The result of the combinatorial analysis showed that the significantly increased microorganisms could encode the enzyme genes for the synthesis of L-Tryptophan, 3-Phosphonooxypyruvate, cis-Aconitate, and Carbamoyl phosphate. In summary, our results demonstrated that *L. Rhamnosus* GG ATCC53103 and *L. Plantarum* JL01 could stimulate the expression of skeletal muscle protein synthesis genes of weaned piglets by modulating the structure of the gut microbiota and its metabolites, thereby improving nitrogen metabolism in weaned piglets.

## 1. Introduction

The gastrointestinal microbial community is diverse and vibrant and plays a crucial role in the health and nutrition of the host (Roager and Licht, 2018). In previous studies, nitrogen metabolism and utilization were generally considered the role of digestive enzymes in the digestive tract. However, gut microbes are rich in functional genes that encode amino acid metabolism and protein synthesis. *Prevotella ruminicola, Butyrivibrio fibrisolvens, Mitsuokella multiacidas*, and *Streptococcus bovis* can secrete highly active dipeptidyl peptidase and dipeptidase for digestion and absorption of proteins. Therefore, gut microbes play a vital role in the metabolism of amino acids (Krajmalnik-Brown et al., 2012; Zhao et al., 2019). It has been reported that small intestinal microbes can use various nutrients in the diet, endogenous secreted proteins, and circulating urea in the intestine to synthesize amino acids (Bergen and Wu, 2009). A study showed that microbes in the small intestine can extensively metabolize various amino acids, with a metabolic rate of more than 50% (Yang et al., 2014). The isotope labeling method can better confirm this conclusion. In a report on lysine, lysine was synthesized by intestinal microorganisms and utilized by the body (Metges, 2000). Although the contribution rate may be small, it is still not negligible in the case of limited intake (Matthews, 2020). Moreover, various amino acids synthesized by intestinal microorganisms may promote muscle metabolism as regulatory factors (Lin et al., 2017). Such as tryptophan stimulates the mTOR pathway in muscle cells, promoting gene expression involved in myofibril synthesis (Dukes et al., 2015).

It has been reported that intestinal microorganisms use proteins to produce a large number of microbial metabolites, some of which are intermediate products and others are final products (Bishu, 2016). Some of these bacterial metabolites are absorbed by intestinal epithelial cells and have a positive effect. Unabsorbed bacterial metabolites are transported to the portal vein and exert physiological effects on the liver and peripheral organs (Davila et al., 2013). For example, tryptophan is metabolized by microorganisms to produce indole and its derivatives (Roager and Licht, 2018), which may induce intestinal endocrine cells to secrete glucagon-like peptide 1, inhibit gastric secretion and peristalsis, and promote satiety (Jardon et al., 2022). The gut microbiota metabolizes and produces many of the vitamins the body needs, especially folic acid and vitamin B_12_ (LeBlanc et al., 2013), which may improve muscle anabolism (Kuo et al., 2007). Therefore, there is a close relationship between gut microbes and nitrogen metabolism.

Weaning stress disrupts the gut microbial balance and reduces mammalian absorptivity for amino acid and protein synthesis (Wang et al., 2019; Yi et al., 2019). Probiotics have been shown to balance gut microbes and affect the body's absorption of amino acids (Utzschneider et al., 2016; Geng et al., 2021). Furthermore, probiotics have beneficial effects on growth performance and nitrogen metabolism during weaning in mammals (Khanian et al., 2019; Wang and Kim, 2021). Our previous study showed that *L. rhamnosus* GG ATCC53103 and *L. plantarum* JL01 improved the growth performance and intestinal immune function of weaned pigs (Geng et al., 2021). However, the effects of *L. rhamnosus* GG ATCC53103 and *L. plantarum* JL01 on the nitrogen metabolism of weaned pigs were still unclear. Therefore, the present study explored the effects of feeding *L. rhamnosus* GG ATCC53103 and *L. plantarum* JL01 on nitrogen metabolism and gut microbes in weaned piglets and used metagenomics to analyze the role of dominant flora. Finally, metagenomics and metabolomics are linked to explore the mechanism of its impact on nitrogen metabolism.

## 2. Materials and methods

### 2.1. Ethics statement

All animal experimental procedures were approved by the Ethics Committee of Jilin Agricultural University (Changchun, Jilin, China).

### 2.2. Animals and experimental design

Forty-eight 35-day-old “Yorkshire-Landrace-Duroc” weaned piglets (average 11.59 kg) were randomly divided into four diet groups (n = 4 each, i = 3 each). Every three piglets are kept in a cage. After a 7-day acclimation period, a 28-day animal experiment was started. The probiotics were cultured in MRS Medium at 37°C for 18h. The control group was fed the basal diet of the National Research Council (2012) nutritional requirements, and each piglet was fed 10 mL of MRS without bacteria per day. The experimental group was fed the basal diet with MRS containing probiotics, and the dose of probiotics per pig per day was included 10 mL containing *L. plantarum* JL01 (1 × 10^9^ CFU/mL Lac group), *L. rhamnosus* GG ATCC53103 (1 × 10^9^ CFU/mL, Rha group) or both probiotics (0.5 × 10^9^ CFU/mL *L. plantarum* JL01 and 0.5 × 10^9^ CFU/mL *L. rhamnosus* GG ATCC53103, mix group). Probiotics or MRS were mixed into a small amount of the basic diet and then fed to the piglets separately with a spoon to ensure that all weaned piglets would eat the whole dose. All piglets can get free water and feed. The composition of the diet is shown in Table 1.

**Table 1:** Composition and nutritional level of the diets.

**Ingredient composition (%)**	**Content**
Corn	67
Soybean meal	15
Whey powder	4.3
Fish meal	5.3
Expanded soybean	3.8
Soybean oil	1.3
Lysine	0.58
Methionine	0.19
Threonine	0.21
Tryptophan	0.05
CaHPO4	0.55
Limestone	0.42
Salt	0.3
Vitamin mineral premix^a^	1
Total	100
Nutritional levels^b^	
DE (MJ/kg)	14.63
Crude protein (%)	16.98
Calcium (%)	0.70
Available phosphorus (%)	0.33
SID Lysine (%)^c^	1.23
SID Methionine (%)	0.68
SID Threonine (%)	0.73
SID Tryptophan (%)	0.2

a Premix provides the following nutrients per kg of diet: Co,1 mg; Cu,150 mg; Fe,150 mg; Zn, 120 mg; Mn, 80 mg; I,0.3 mg; Se,0.3 mg; nicotinamide,10 mg; calcium pantothenate, 5 mg; folic acid, 0.4 mg; D-biotin, 0.05 mg; vitamin A, 38000000 IU; vitaminD3, 8,000,000 IU; vitamin E,90000 IU; vitamin K3, 1 mg; vitamin B1,1 mg; vitamin B2, 2 mg; vitamin B6, 1.2 mg; vitamin B12, 0.01 mg; Antioxidant 0.02 mg.

b Nutrition levels, in addition to crude protein (CP), are measured values. The rest are calculated values.

c SID: Amino Acid Standard Ileum Digestibility.

### 2.3. Sample collection

On the 23rd day of the rearing period. The metabolic experiments were conducted for 5 days. Using the method of collecting feces and urine, we randomly selected three piglets from each group for the nitrogen metabolism test, fecal weight and urine volume were measured using a separate collection device for feces and urine in metabolic cages, and feces and urine were collected at 8:00 and 16:00 every day (Feyera et al., 2019). Urine samples were added with 10% sulfuric acid for nitrogen fixation and stored at −80°C for testing. We mixed the fecal samples collected every day, kept them in zip lock bags, recorded the weight, took out 200 g of fecal samples, quickly added 10% sulfuric acid to fix nitrogen, baked them at 60 °C to constant weight, then pulverized, and stored at −80 °C refrigerator for testing. On the morning of the 28th day, three weaned piglets were randomly selected from each group to collect intestinal samples, and a 3% sodium barbital solution was used as anesthesia for weaned piglets. The jejunum and ileum were dissected, and the contents of the jejunum and ileum were carefully squeezed out, and then, chyme was collected in sterile cryogenic vials. Afterward, 10 mL of hepatic portal vein blood was collected and placed in a blood collection tube containing heparin sodium. After centrifugation, plasma layers were extracted and distributed into 1.5 mL cryopreservation tubes. Afterward, the skeletal muscle (gluteus maximus) of piglets was quickly collected with a sterile EP tube and quickly frozen in liquid nitrogen. The samples were stored in a −80°C refrigerator.

### 2.4. Nitrogen metabolism experiment

The nitrogen content in feed and feces was measured using Nitrogen determination apparatus (FOSS Kjeltec 8420 Kjeldahl +nitrogen determination apparatus, FOSS [Technology and Trade Co. Ltd., Beijing, China]). The formulas used were as follows: nitrogen emission (g/d) = fecal nitrogen (FN) (g/d) + urine nitrogen (UN) (g/d), nitrogen apparent digestibility (%) = [(nitrogen intake (NI) - FN)/NI] × 100%, nitrogen deposition rate (%) = [(NI-FN-UN)/NI] × 100%, and nitrogen utilization rate (%) = [(NI-FN-UN) / (NI-FN)] × 100%.

### 2.5. Real-time quantitative PCR

Total RNA from the muscle tissue was extracted using TRIzol. The steps for RNA-reverse transcription and RT-qPCR were the same as before (Ding et al., 2020). PrimeScript™ RT reagent kit and SYBR^®^ Premix Ex T aq™ II were used for reverse transcription and RT-qPCR processes, respectively. RT-qPCR analysis was conducted using the Agilent Stratagene Mx3005P qPCR system (Agilent Technologies, Inc.). These RT-qPCR assays were run in triplicate for each sample, and the results were normalized to the expression of β-actin. Expression was analyzed using 2–ΔΔCt comparison method. Primer sequences are provided in Table 2.

**Table 2:** Parameters of primer for real-time PCR.

**Genes**	**Primer sequences (5'-3')**	**Product size (bp)**	**Annealing temperature**
mTOR	Forward GGCGATAGACACCCATCTAACC	83	62°C
	Reverse ACCTCAAAGCAGTCCCCAAA		
S6K	Forward TCCAATACGACAGCCGAACT	163	58°C
	Reverse TCACCTTGCAGGATGCTCAC		
4EBP1	Forward GAAGTTCCTAATGGAGTGTCGG	191	58°C
	Reverse TGTCCATCTCAAACTGTGACTCTT		
β-actin	Forward TGGTTCTGGGCTCTGTAAGG	190	57°C
	Reverse GATGCCGTGTTCTATTGGGT		

###  2.6. DNA extraction and 16S rRNA sequencing

Gut chyme microbial DNA was extracted using the TIANamp Stool DNA kit (Tiangen, Beijing, China).

The sequences of 16S rDNA genes were analyzed using the Illumina HiSeq™ 2500 sequencing platform. The raw data were filtered using the FASTP software. All samples were clustered, and the effective sequence similarity ≥ 97% was clustered into operation classification units (OTUs). The OTU sparsity curve and rank abundance curve were plotted in QIIME. An inter-group Venn analysis was performed in R (version 3.4.1) to identify unique and common otus. We use QIIME to calculate Chao1, Simpson, etc. α Diversity Index.

### 2.7. Sample preparation for LC-MS analysis

Portal vein plasma samples weighing 50 mg were carefully measured and transferred into an EP tube. Subsequently, the EP tube was filled with the solvent (acetylene-methane-water, 2: 2: 1, including internal standards). After rotating for 30s at an average speed of 45 Hz, the sample was subjected to a 4-min ice water bath. Then, it was ultrasonicated for 5min. The average ultrasound is repeated three times and then stored at −20 °C for 1 h and for 15 min for 12, 000 RPM and 4 °C. The obtained Shangqing was transferred to the LC-MC bottle, analyzed in UHPLC-QE or orBITRAP MS, and mixed with the equivalent of all samples to clear the quality control (QC) sample.

### 2.8. Functional analysis

We used the *T*-test as a variable analysis to screen the different metabolites, and the VIP threshold was set to 1, and the differences between the two groups of the metabolites of a *P-*value of < 0.05 and a VIP of ≥ 1 were selected. The metabolites were enriched in the KEGG metabolic pathway for enrichment analysis. Compared with the entire background, the enrichment analysis determined the significant enrichment of metabolites or signal transition channels in the difference.

### 2.9. Sequence analysis

We used the Nextera XT DNA Library to prepare the library for the macro-base group and sequence the Illumina Hiseq platform. We also used Metaphlan (V.2.2) (Truong et al., 2015) and Humann2 to analyze macroscopic group data. Then, we use the Unigene to query the enzyme committee (EC) database through Diamond software. NCBI's RefSeq database was used to build a microbial index with significant differences in pigs annotated by Metaothello.

### 2.10. Statistical analysis

The results were expressed as the mean ± SD. Statistical analysis was performed using Prism software (GraphPad Software, San Diego, CA) to carry out one-way ANOVA followed by Bonferroni *post-hoc* analysis. Values of *p* < *0.05* were considered significant. Different microbial, genetic functions, metabolites, and metabolic channels were drawn through Adobe Illustrator software (USA).

## 3. Results

### 3.1. Nitrogen metabolism

The effects of *L. plantarum* JL01 and *L. rhamnosus* GG ATCC53103 on the nitrogen metabolism of weaned piglets are shown in Table 3. Compared to the Con group, the ingested nitrogen of the piglets in the Rha group, Lac group, and mix group was significantly increased (*P* < 0.05), fecal nitrogen, urinary nitrogen, and total nitrogen emissions showed a decreasing trend, the apparent nitrogen digestibility of Rha and Lac groups tended to increase; the apparent nitrogen digestibility of the mix group increased by 1.88% (*P* < 0.05), and the nitrogen deposition rate increased respectively increased by 4.17, 4.49, and 5.65% (*P* < 0.05), and the nitrogen utilization rate increased by 2.82, 3.23, and 3.70%, respectively (*P* < 0.05).

**Table 3:** Effects of *L. plantarum* JL01, *L. rhamnosus* GG ATCC53103 on nitrogen metabolism of weaned piglets.

	**Con**	**Rha**	**Lac**	**Mix**	***P*-value**
Nitrogen intake (g/d)	31.25 ± 0.80^a^	34.81 ± 0.60^b^	35.48 ± 0.76^b^	35.99 ± 0.46^b^	< 0.001
Fecal nitrogen (g/d)	4.50 ± 0.17	4.63 ± 0.16	4.74 ± 0.12	4.61 ± 0.11	0.310
Urine Nitrogen (g/d)	4.95 ± 0.18	4.90 ± 0.32	4.89 ± 0.29	4.87 ± 0.14	0.970
Nitrogen emissions (g/d)	9.46 ± 0.17	9.53 ± 0.25	9.63 ± 0.19	9.48 ± 0.17	0.674
Nitrogen apparent digestibility (%)	85.58 ± 0.84^a^	86.70 ± 0.68^ab^	86.63 ± 0.50^ab^	87.19 ± 0.24^b^	0.0592
Nitrogen deposition rate (%)	69.71 ± 0.76^a^	72.62 ± 0.82^b^	72.84 ± 0.56^b^	73.65 ± 0.38^b^	< 0.001
Nitrogen utilization rate (%)	81.46 ± 0.21^a^	83.76 ± 0.87^b^	84.09 ± 0.42^b^	84.47 ± 0.33^b^	< 0.001

Con: control group; Rha: *L. rhamnosus* GG ATCC53103; Lac: *L. plantarum* JL01; Mix: *L. plantarum* JL01 and *L. rhamnosus* GG ATCC53103. Data are expressed as means ± SD. Dissimilar letters represent significant differences among different groups (P < 0.05).

### 3.2. Gene expression of skeletal muscle protein deposition

**Figure 1:**
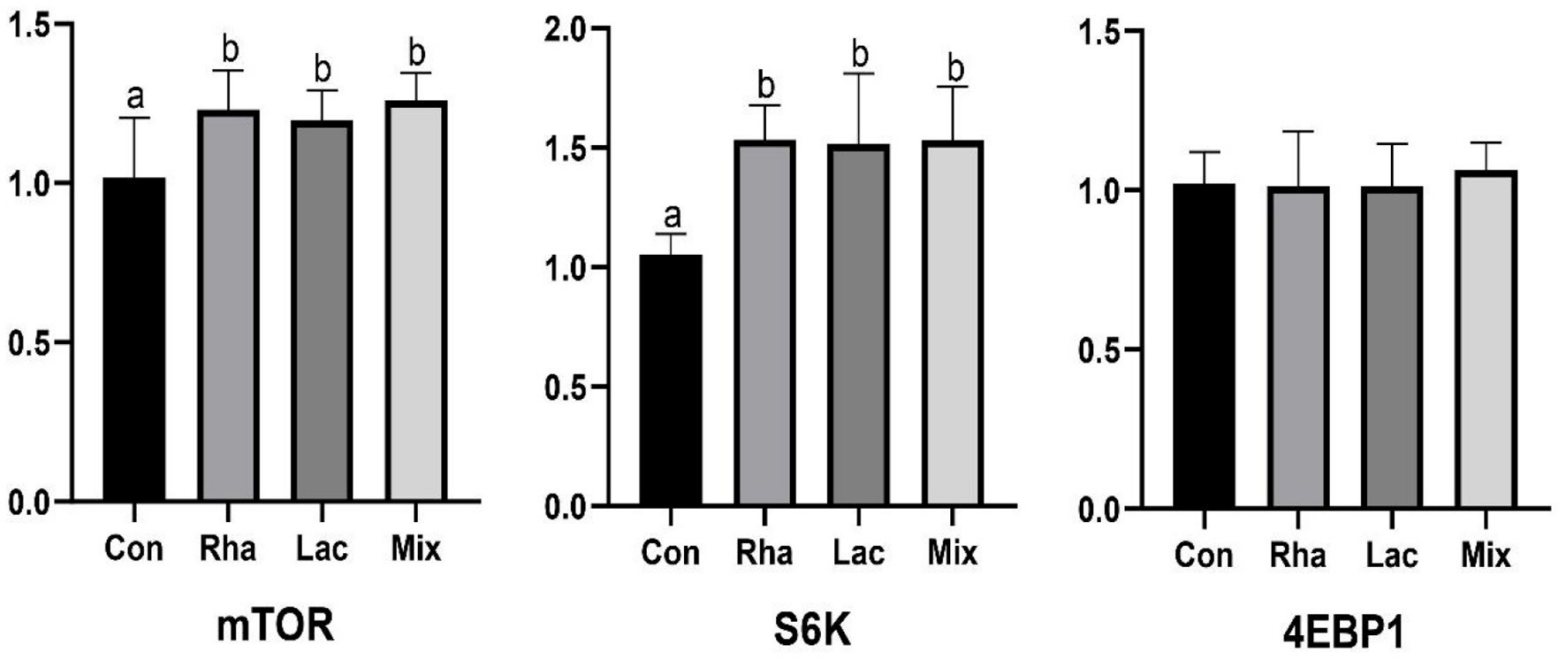
The relative expression of mTOR, S6K, 4EBP1 mRNA of the dorsal longest muscle of weaned piglets. Con: control group; Rha: *L.rhamnosus* GG ATCC53103; Lac: *L. plantarum* JL01; Mix: *L. plantarum* JL01 and *L. rhamnosus* GG ATCC53103. Data are expressed as means ± SD. Dissimilar letters represent significant differences among different groups (*P* < 0.05).

As shown in Figure 1, the relative expression of mTOR and S6K mRNA in the skeletal muscle of piglets in the Rha, Lac, and mix groups was significantly increased compared with the Con group (*P* < 0.05).

### 3.3. Data acquired from high-throughput sequencing and alpha-diversity measurements

**Figure 2:**
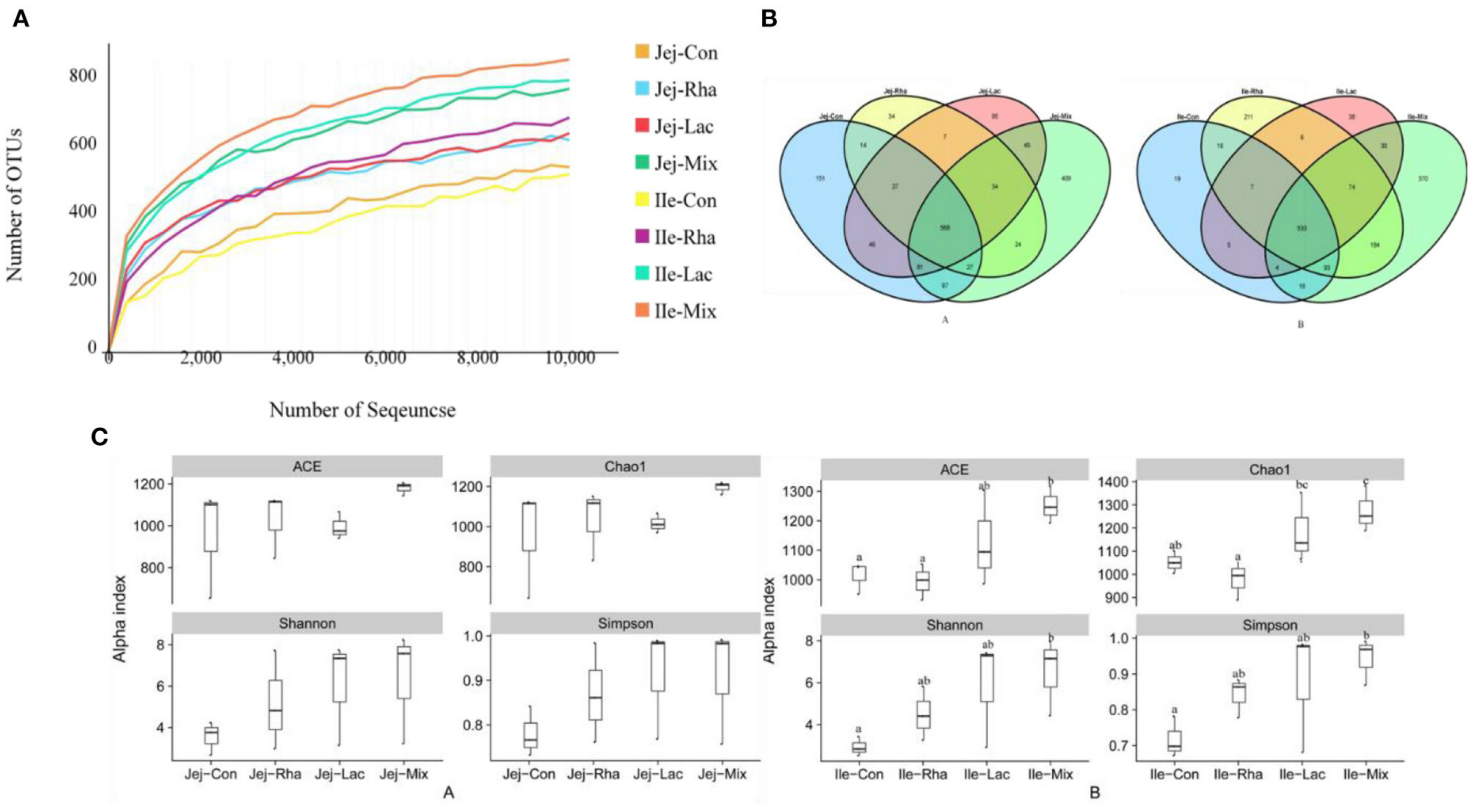
**(A)** Rarefaction curves of OTUs from microbiota in the jejunum and ileum of weaned piglets. **(A)** OTU comparative analysis of intestinal microbiota of weaned piglets **(A)** jejunum **(B)** ileum. **(C)** Box pattern of microbial Alpha diversity index of weaned piglets **(A)** jejunum **(B)** ileum; Con: control group; Rha: *L. rhamnosus* GG ATCC53103; Lac: *L. plantarum* JL01; Mix: *L. plantarum* JL01 and *L. rhamnosus* GG ATCC53103. Data are expressed as means ± SD. Dissimilar letters represent significant differences among different groups (*P* < 0.05).

A total of 2, 800, 880 sequences were obtained in this experiment, and the original sequences were filtered, and finally, 2, 591, 938 valid sequences were obtained, with an average of 128, 160 valid sequences per sample for subsequent analysis. Microbial diversity is the diversity in a particular habitat or ecosystem and uses two parameters, species richness, and evenness, to reflect the diversity and abundance of microorganisms (Grice et al., 2009). Chao1 and ACE indices were used to estimate species richness information of samples, and Simpson and Shannon were used to estimate species diversity information (Segata et al., 2011). The effects of *L. plantarum* JL01 and *L. rhamnosus* GG ATCC53103 on the microbial diversity of the small intestine of weaned piglets are shown in Figure 2. Compared with the Con group, the Shannon and Simpson indices of the Rha, Lac, and mix groups showed an increasing trend in the jejunum but were insignificant. In the ileum, the ACE, Chao1, Shannon, and Simpson indexes of piglets in the mix group were significantly increased compared with the Con group (*P* < 0.05), while the uniformity and abundance of other intestinal contents were not significant.

### 3.4. Changes in gut microbiota

**Figure 3:**
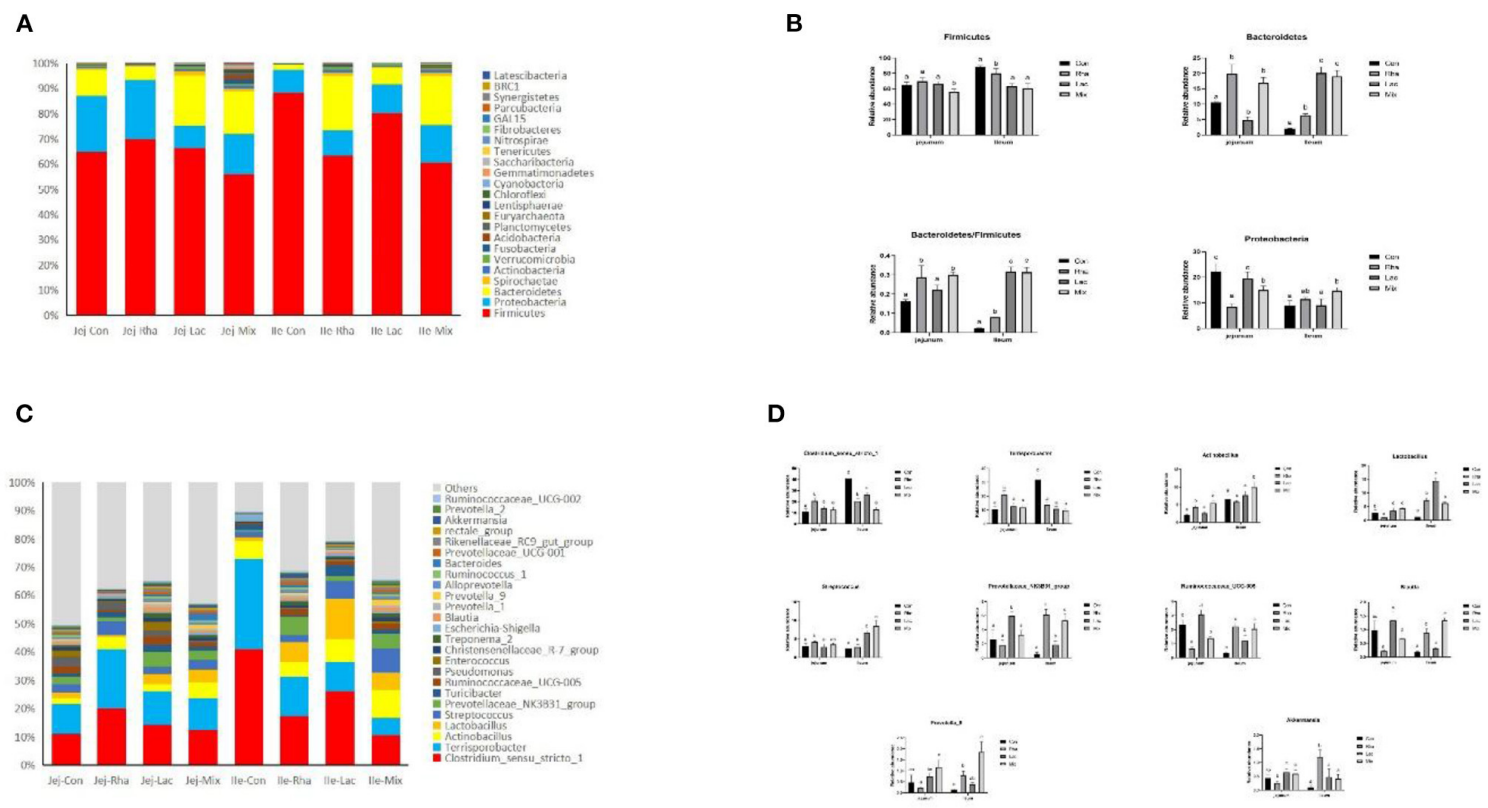
**(A)** The top 10 most abundant phyla are presented. **(B)** Bacterial composition differences in the small intestine at the phylum level. Con: control group; Rha: *L. rhamnosus* GG ATCC53103; Lac: *L. plantarum* JL01; Mix: *L. plantarum* JL01 and *L. rhamnosus* GG ATCC53103. Data are expressed as means ± SD. Dissimilar letters represent significant differences among different groups (*p* < 0.05). **(C)** Relative abundances of the top ten genera. **(D)** Bacterial composition differences in the small intestine at the genus level. Con: control group; Rha: *L. rhamnosus* GG ATCC53103; Lac: *L. plantarum* JL01; Mix: *L. plantarum* JL01 and *L. rhamnosus* GG ATCC53103. Data are expressed as means ± SD. Dissimilar letters represent significant differences among different groups (*P* < 0.05).

The microbial composition of the gut contents of weaned piglets was analyzed. Figure 3A shows the 10 most abundant phyla among all phyla. *Firmicutes, Proteobacteria*, and *Bacteroidetes* are the common dominant phyla in each treatment group, and their relative abundances together account for the bacterial abundances in each treatment group. They accounted for more than 93% of the total bacteria. As shown in Figure 3B, compared with the Con group, in the jejunum, the *Firmicutes* in the mix group were significantly decreased (*P* < 0.05). The relative abundance of *Bacteroidetes* significantly increased (*P* < 0.05), and the relative abundance of Proteus significantly decreased (*P* < 0.05) in the Lac and mix groups. *Firmicutes*/*Bacteroidetes* were significantly increased in piglets in the Lac and mix groups (*P* < 0.05). In the ileum, the relative abundance of *Firmicutes* in the Rha and mix groups was significantly lower than those in the Con group (*P* < 0.05). The relative abundance of *Bacteroidetes* increased significantly in Rha, Lac, and mix groups (*P* < 0.05). *Firmicutes*/*Bacteroidetes* were significantly increased in Rha, Lac, and mix groups (*P* < 0.05).

At the genus level, 181 genera were identified from all samples. The relative abundances of the top 25 genera are shown in Figure 3C. As shown in Figure 2D, compared with the control group, the relative abundance of *Lactobacillus, Actinobacillus*, and *Prevotella*_9 in the mix group increased significantly (*P* < 0.05), and the relative abundance of *Ruminococcaceae*_UCG-005 decreased significantly (*P* < 0.05). The relative abundances of *Ruminococcaceae*_UCG-005, *Actinobacillus, Streptococcus, Blautia*, and *Prevotella*_9 increased significantly (*P* < 0.05), while the relative abundances of *Clostridium*_sensu_stricto_1 and *Terrisporobacter* decreased significantly (*P* < 0.05). Rha group in the jejunum, including *Clostridium*_sensu_stricto_1, *Terrisporobacter*, and *Actinobacillus*, were found to be significantly different. The relative abundance of *Lactobacillus, Ruminococcaceae*_UCG-005, and *Streptococcus* decreased significantly (*P* < 0.05). Conversely, in the ileum, the relative abundance of the Rha group, *Prevotellaceae*_NK3B31_group, *Lactobacillus, Ruminococcaceae*_UCG-005, and *Akkermansia*, increased significantly (*P* < 0.05). Additionally, the relative abundance of *Clostridium*_sensu_stricto_1 and *Terrisporobacter* decreased significantly (*P* < 0.05). The relative abundance of the Lac group, including *Prevotellaceae*_NK3B31_group, *Lactobacillus, Ruminococcaceae*_UCG-005, *Prevotella*_9, showed a significant increase (*P* < 0.05). Similarly, in the ileum, the relative abundance of the Lac group, comprising *Prevotellaceae*_NK3B31_group, *Lactobacillus, Ruminococcaceae*_UCG-005, and *Streptococcus*, also increased significantly (*P* < 0.05). In contrast, the relative abundance of *Clostridium*_sensu_stricto_1 and *Terrisporobacter* exhibited a significant decrease (*P* < 0.05).

### 3.5. Small intestine differentiated microbial and functional gene network map

**Figure 4:**
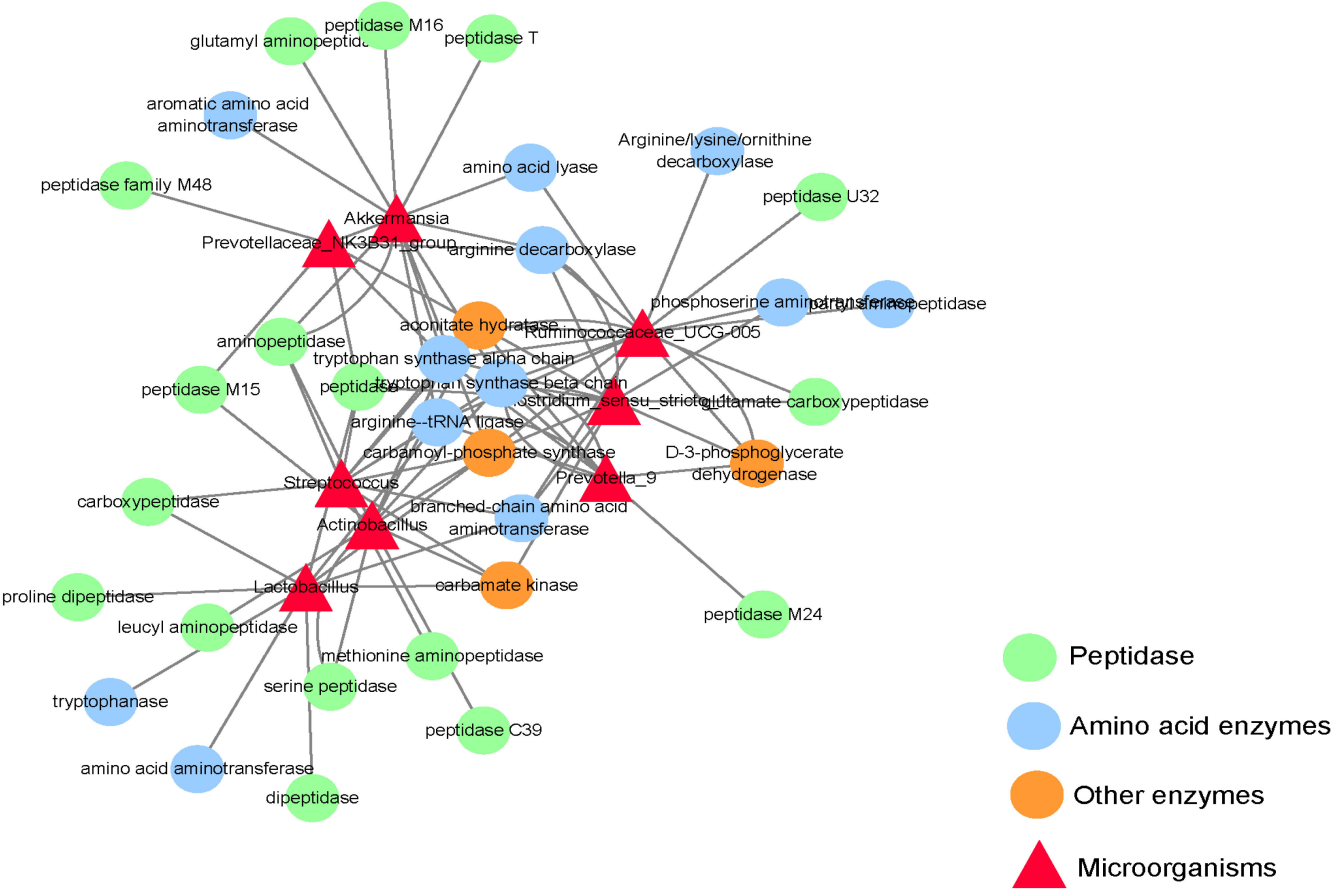
Network diagram of differential microbes and functional genes in the small intestine of the weaned piglets.

This experiment only focuses on the differentiated microorganisms in the small intestine and the functional genes encoding amino acid metabolism and protein synthesis. As shown in Figure 4, *Clostridium_ sensu_ stricto_ 1* and *ruminocaceae_ Ucg-005* have a gene encoding d-3-phosphoglycerate dehydrogenase; *Actinobacillus, Clostridium, Lactobacillus*, and *Streptococcus* have genes encoding carbamate kinase and Carbamoyl phosphate synthase; *Akkermansia* and *Ruminococcus* have genes encoding Carbamoyl phosphate synthase; *Actinomycetes, Clostridium, Prevotellaceae_ NK3B31_ Group, Prevotella_ 9, Rumencocci* and *Streptococcus* have genes encoding aconitate hydratase; *Actinomycetes, Klebsiella, Streptococcus, Rumencocci, Prevotellaceae_ NK3B31_ Group, Prevotella_ 9* and *Clostridium* has a gene encoding tryptophan synthase.

### 3.6. Metabolomic profiling of portal vein

In this test, no significant peaks were detected in the blank sample, indicating that there was no cross-contamination between samples in this test. Substance identification was conducted using a self-written R program package and a self-built secondary mass spectrometry database. The second-order mass spectrometry matching degree cutoff = 0.2. A total of 11, 875 metabolites were obtained from the detection results of this study, and 617 substances were identified by the secondary spectrum.

**Figure 5:**
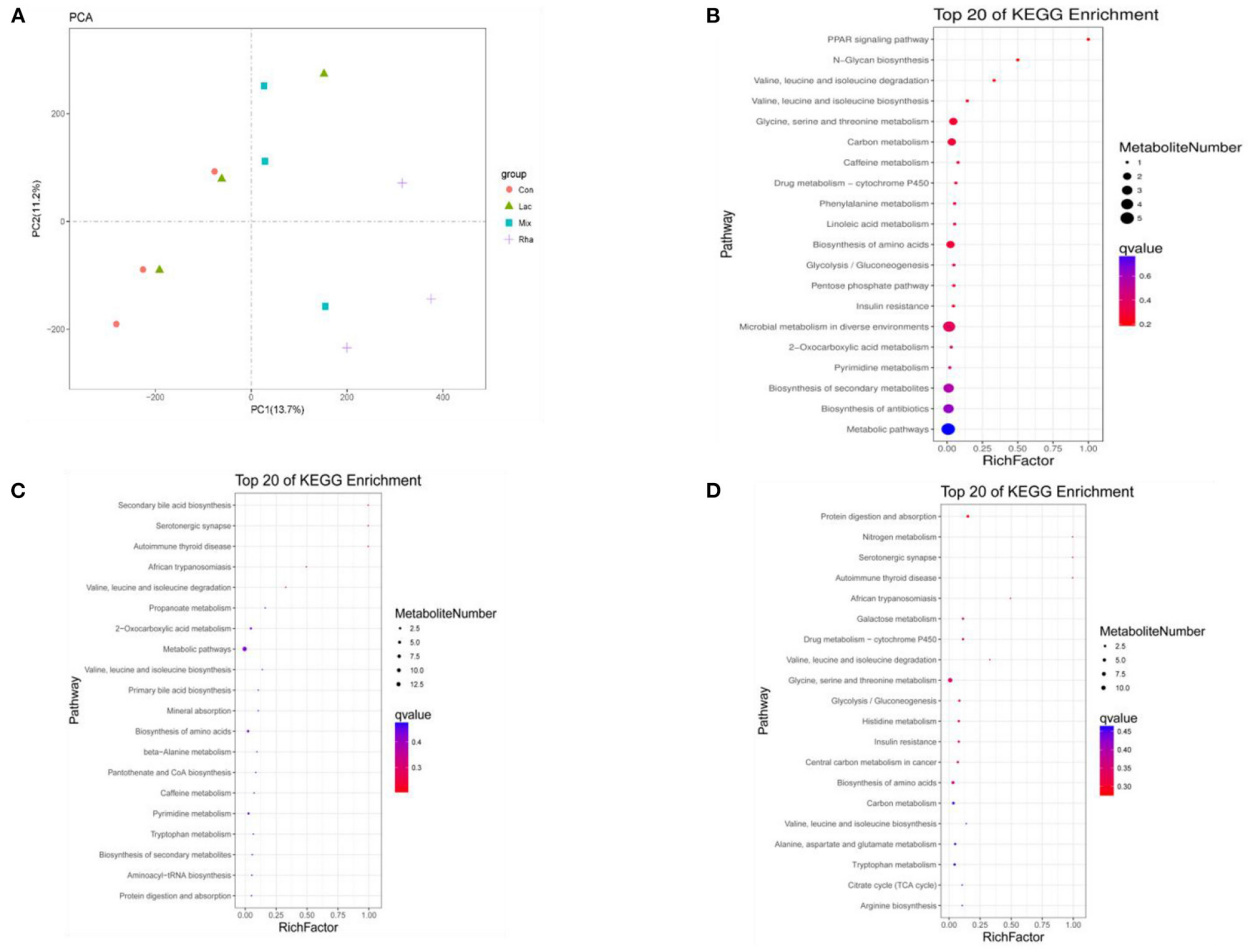
**(A)** PCA score map of metabolites in plasma of hepatic portal vein. Distribution of enriched KEGG pathway of differential metabolites in plasma of hepatic portal vein **(B)** Con vs. Rha **(C)** Con vs. Lac **(D)** Con vs. Mix.

As shown in Figure 5A, each treatment group had apparent separation compared with the control group, indicating that feeding *L. rhamnosus* and *L. plantarum* had a significant effect on the plasma metabolites of the hepatic portal vein of weaned piglets. In this study, the differential metabolites related to nitrogen metabolism were selected for analysis. As shown in Table 4, in the portal vein metabolites, compared with Con, the differential metabolites produced by the Rha group were 3-Phosphonooxypyruvate (*P* < 0.05), the differential metabolites produced by the Lac group were L-Tryptophan (*P* < 0.05), and the differential metabolites produced by the mix group were L-Tryptophan, 3-Phosphonooxypyruvate, cis-Aconitate and Carbamoyl phosphate (*P* < 0.05). As shown in Figures 4A–D, after the metabolites were enriched, the first 20 paths with the lowest enrichment q value were selected to draw the map. This experiment only focused on the metabolic pathway of nitrogen nutrients. The results showed that the differential metabolite 3-phosphodeoxypyruvate enriched in the portal vein of weaned piglets produced by feeding the mixed bacteria of *L. rhamnosus* and *L. plantarum* was glycine, serine, and threonine metabolism pathway; the metabolic pathway enriched by the differential metabolite L-Tryptophan is the tryptophan metabolic pathway; the metabolic pathway enriched by the differential metabolite cis-Aconitate is the Citrate cycle (TCA cycle) pathway; The metabolic pathway for the differential metabolite Carbamoyl phosphate is the Arginine biosynthesis pathway.

**Table 4:** Differential metabolites in plasma of hepatic portal vein.

**Groups**	**Metabolites name**	**Treatment group Trend**	**Pathway**	***P-*value**	**VIP**
Con vs. Rha	3-Phosphonooxypyruvate^*^	↑	Glycine, serine, and threonine metabolism; Biosynthesis of amino acids	0.05	1.47
Con vs. Mix				0.02	1.35
Con vs. Mix	Carbamoyl phosphate^*^	↑	Nitrogen metabolism; Biosynthesis of amino acids; Alanine, aspartate, and glutamate metabolism; Arginine biosynthesis	0.01	1.33
Con vs Mix	cis-Aconitate^*^	↑	Citrate cycle (TCA cycle)	0.03	15.50
Con vs. Lac	L-Tryptophan^*^	↑	Protein digestion and absorption; Biosynthesis of amino acids; Glycine, serine, and threonine metabolism; Tryptophan metabolism; Phenylalanine, tyrosine, and tryptophan biosynthesis; Biosynthesis of secondary metabolites	0.03	7.14
Con vs. Mix				0.01	7.05

Rha: *L. rhamnosus* GG ATCC53103; Lac: *L. plantarum* JL01; Mix: *L. plantarum* JL01 and *L. rhamnosus* GG ATCC53103. Data are expressed as means. ^*^Represent significant differences among different groups (P < 0.05) and VIP ≥ 1.

### 3.7. Relationship between the intestinal microbiome and non-targeted metabolomics

**Figure 6:**
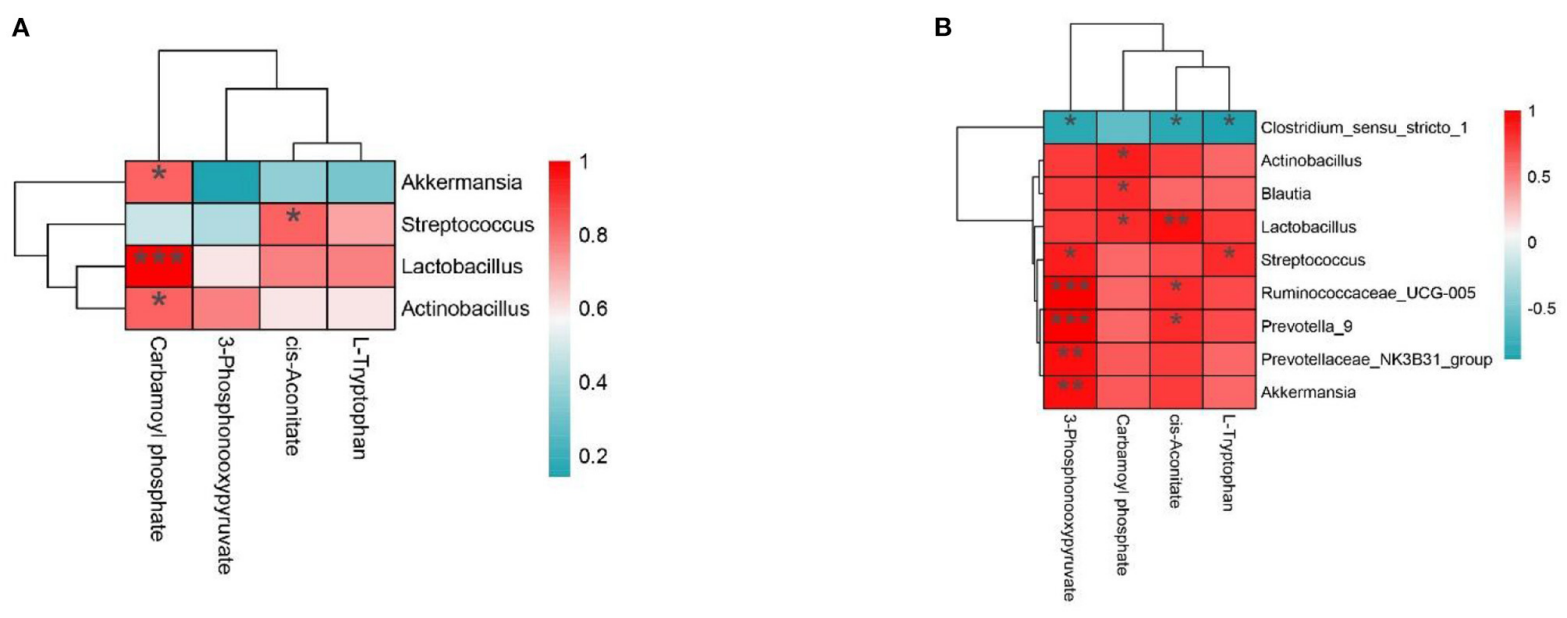
**(A, B)** The interaction between intestinal flora and portal vein metabolome in the Con group vs. Mix group.^*^P < 0.05, ^**^P < 0.01, and ^***^P < 0.001 indicate significant differences between the microbiota and metabolites. Blue represents positive correlations; red represents negative correlations.

To further study the relationship between intestinal microflora and metabolites, we visualized the correlation between jejunum and ileum microflora and metabolites and carried out Spearman correlation analysis on the relative abundance of different microbial species and metabolites. As shown in Figure 6A, in the jejunum, L-Tryptophan and cis-Aconitate were positively correlated with *Streptococcus, Lactobacillus*, and *Actinobacillus* and negatively correlated with *Akkermansia*. Carbamoyl phosphate was positively correlated with *Akkermansia, Lactobacillus*, and *Actinobacillus* and negatively correlated with *Streptococcus*. 3-Phosphonoxypyruvate was positively correlated with *Lactobacillus* and *Actinobacillus* and negatively correlated with *Akkermansia* and *Streptococcus*. As shown in Figure 6B, in the ileum, we found 3-Phosphonooxypyruvate, Carbamoyl phosphate, cis-Aconitate, L-Tryptophan and *Actinobacillus, Blautia, Lactobacillus, Streptococcus, Ruminococcaceae_UCG-005, Prevotella_9, Prevotellaceae_NK3B31_group*, and *Akkermansia* were positively correlated and negatively correlated with *Clostridium_sensu_stricto_1*.

## 4. Discussion

During pig production, approximately 46% of nitrogen was retained in the animal's body, and the remaining 54% was excreted mostly in feces and urine. Therefore, improving the efficiency of converting plant protein feed into animal protein could reduce pollution caused during pork production and possibly increase economic efficiency (Millet et al., 2018). The small intestine is the main site of absorption of dietary protein. Most of the nitrogenous nutrients in the diet are digested in the small intestine and converted into amino acids or small peptides; the unabsorbed part is directly excreted with the feces, and part of the absorbed part is used by the animal body to become deposited nitrogen, and the other part is passed through the ornithine cycle to generate urea nitrogen and excreted in the urine (Zhao et al., 2020). At the same time, gut microbes also play an important role. They use nitrogen sources in the gut to synthesize amino acids and participate in nitrogen metabolism (van der Wielen et al., 2017). Through the analysis of nitrogen metabolism, the preliminary situation of nitrogen utilization in the body is reflected. In the present study, we found that the combined feeding of *L. plantarum* JL01 and *L. rhamnosus* GG ATCC53103 had the best effect, increasing the apparent nitrogen digestibility by 1.31%, the nitrogen deposition rate by 5.65%, and the nitrogen utilization rate by 3.70% in weaned piglets and was consistent with previous reports (Zhu et al., 2017).

Intestinal microorganisms play an important role in the digestive system of animals (Sun et al., 2019). It has been reported that the higher the diversity of intestinal microorganisms, the higher the stability of the body's intestinal microecosystem, thus promoting the growth of animals and the absorption and utilization of nutrients (Cani and Delzenne, 2009; McCann et al., 2017). Previous studies have shown that dietary supplementation with probiotics improves the diversity of gut flora, which is consistent with our results (Sun et al., 2023). Moreover, a large number of amino acids in the small intestine are transformed, metabolized, and utilized by intestinal microorganisms, which not only maintains the composition and quantity of the microorganisms themselves but also produces metabolites that regulate the physiological functions of the host (Nicholson et al., 2012), and could also utilize various substrates to synthesize new microbial amino acids (Torrallardona et al., 2003; Metges and Petzke, 2005). In our study, we found that co-administration of the two probiotics significantly improved the abundance of *Prevotella, Streptococcus, Actinomyces*, and *Klebsiella* in the jejunum and ileum chyme significantly increased in piglets. At present, there are few articles on the relationship between small intestinal microorganisms and amino acids, so we detected the functional genes of small intestinal microorganisms through the macrogenome to predict the relationship between small intestinal microorganisms and amino acids. In our experiment, macrogenomic data showed that *Prevotella, Streptococcus, Actinomyces*, and *Klebsiella* secreted proteinases and alkaline peptidases that hydrolyze proteins and amino acids. This plays an important role in the digestion of mammalian proteins and the absorption of amino acids. *Actinomyces, Klebsiella, Streptococcus, Ruminococcus, Prevotella, Lactobacillus*, and *Clostridium* all have genes encoding various peptidases. *Clostridium, Lactobacillus, Ruminococcus*, and *Streptococcus* have genes encoding branched-chain amino acid aminotransferases. In addition, *Actinomyces, Klebsiella, Streptococcus, Ruminococcus, Prevotella, Lactobacillus*, and *Clostridium* have genes encoding arginase. Previous studies have demonstrated that *Klebsiella* may synthesize aspartic acid, glutamic acid, and tryptophan, *Streptococcus* may synthesize leucine, lysine, and serine, and *Clostridium* may synthesize serine, proline, and valine, which aligns with our results (Hooper et al., 2002; Dai et al., 2011). In summary, when the *L. rhamnosus* GG ATCC53103 and *L. plantarum* JL01 are combined, it can increase the number of beneficial bacteria in the pig's intestine and adjust the intestinal microorganisms.

**Figure 7:**
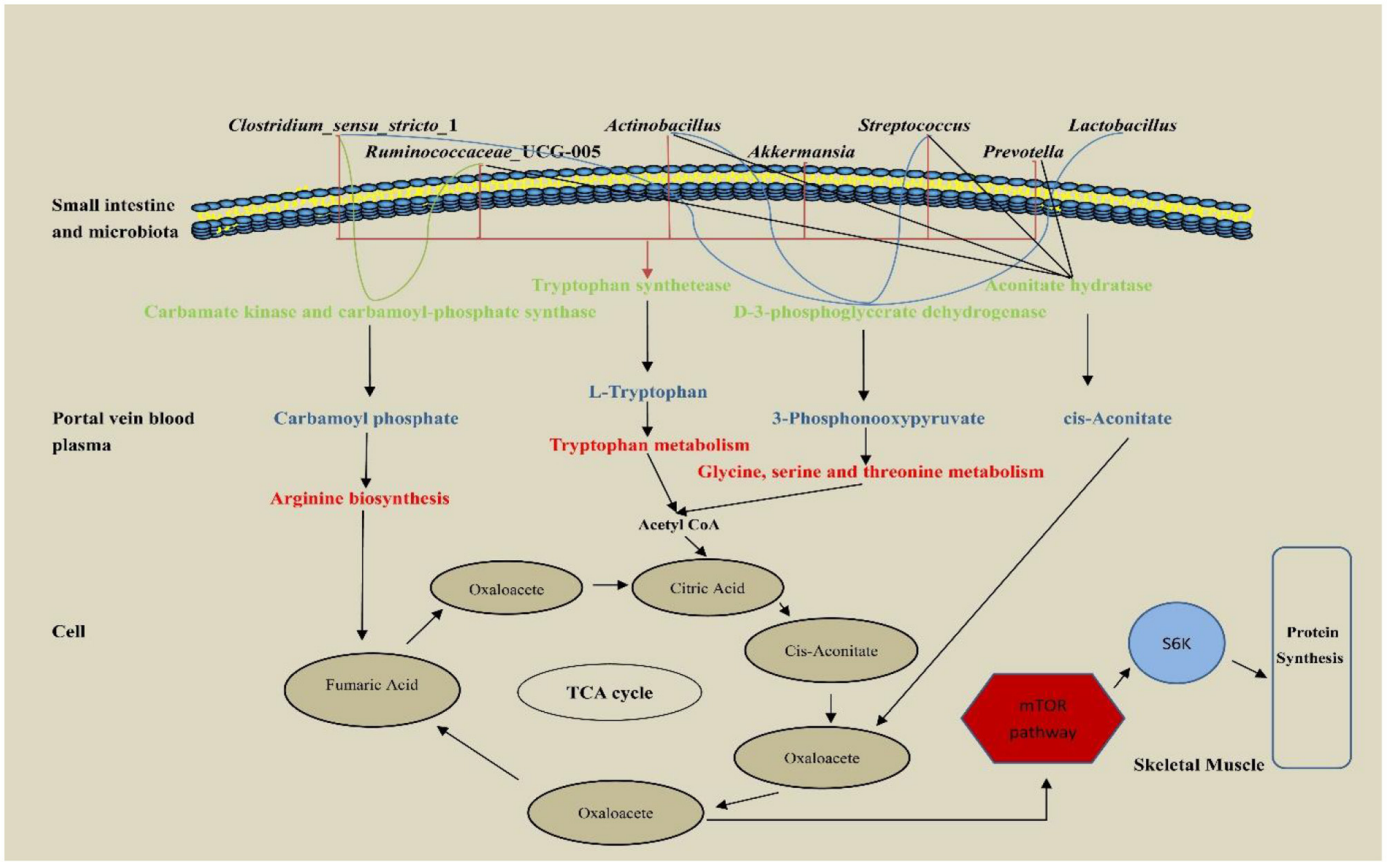
The pathway of hepatic portal vein differential metabolites regulatory protein synthesis of weaned piglets.

The hepatic portal vein is a large vein that enters the liver from the hepatic portal after the mesenteric veins of the digestive tract converge. It is closely related to the metabolism of proteins, lipids, carbohydrates, vitamins, and hormones (Johnson et al., 2016). In this study, portal vein plasma analysis was performed in the Con, Rha, Lac, and mix groups. Compared with the Con group, four important metabolites, 3-Phosphonooxypyruvate, Carbamoyl phosphate, cis-Aconitate, and L-Tryptophan, were detected in the mix group. To detect the relationship between metabolites and intestinal microflora, we analyzed the functional genes of intestinal microflora through macrogenome to analyze the reasons for the increase in metabolites (Figure 7). Our data show that D-3-phosphoglycerate dehydrogenase was encoded by *Clostridium, Prevotella*, and *Ruminococcus*, which is an enzyme that converts 3-phosphoglycerate to phosphopyruvate. The genes of carbamate kinase and carbamoyl phosphate synthase are encoded by *Actinomycetes, Clostridium, Lactobacillus*, and *Streptococcus*, which can convert ammonia to carbamate. Aconitic acid hydratase is encoded by *Actinomycetes, Clostridium, Prevotella, Ruminococcus*, and *Streptococcus*, which can convert citric acid and isocitric acid into cis-Aconitic acid. L-tryptophan is encoded by *Actinomycetes, Klebsiella, Streptococcus, Ruminococcus, Prevotella*, and *Clostridium*, which have genes for tryptophan synthase. Compared with the Con group, these microorganisms were significantly increased in the mix group. In addition, our results showed that most microorganisms were positively correlated with metabolites. Through KEGG enrichment analysis, it was found that cis-Aconitic acid was enriched in the TCA cycle and was an important intermediate product of the TCA cycle. Its increase may promote the TCA cycle and lead to an increase in α-ketoglutarate (AKG). It is well known that AKG plays an important role in protein metabolism and transmembrane transport of amino acids (Wernerman and Hammarqvist, 1999; Zhao et al., 2020) found that adding AKG to a low-protein diet can improve the nitrogen metabolism efficiency of weaned piglets. In addition, the levels of S6K, mTOR, and 4EBP1 mRNA and phosphorylated protein in muscle were higher. In an in vitro study by Cai et al. (2020), it was shown that AKG promotes protein synthesis in C2C12 myotubes through the Akt/mTOR signaling pathway. Our data showed that the relative expression of mTOR and S6K mRNA in skeletal muscle increased significantly. Therefore, cis-Aconitic acid may promote protein synthesis in skeletal muscle by increasing the content of AKG, thereby improving the efficiency of nitrogen metabolism. Moreover, the significantly elevated metabolites 3-Phosphonooxypyruvate and Carbamyl phosphate in the portal vein may also promote protein synthesis by promoting the increase of AKG caused by the TCA cycle. 3-Phosphonooxypyruvate is an intermediate substance in the metabolic pathway of glycine, serine, and threonine and has the function of promoting the metabolism of glycine, serine, and threonine to produce metabolites such as acetyl CoA, propionic acid, and α-aminobutyric acid (Cogo et al., 2018). Acetyl-CoA produced by 3-Phosphonooxypyruvate metabolism may be involved in the TCA cycle by synthesizing citric acid. Carbamoyl phosphate could participate in the urea cycle to generate fumaric acid and participate in the TCA cycle. Tryptophan is a special amino acid that can participate in host protein synthesis, increase host feed intake, and ensure host growth performance (Sterndale et al., 2020). Previous studies have shown that dietary supplementation of moderate tryptophan significantly reduces serum urea and improves growth performance (Ruan et al., 2014; Rao et al., 2021), which is consistent with our results. In addition to producing acetyl-CoA to participate in the TCA cycle, tryptophan can also be used as a raw material for protein synthesis to directly promote protein synthesis.

Interestingly, carbamoyl phosphate may also metabolize arginine to promote protein synthesis. A previous study has shown that arginine is the most abundant nitrogen carrier for protein synthesis in the body (Wu et al., 1999), and in mammals, arginine has the ability to stimulate the secretion of growth factors in the anterior pituitary gland (Flynn et al., 2002), so young animals are more sensitive to arginine requirements that are particularly high (Watanabe et al., 2009). In addition, dietary arginine supplementation can improve nitrogen use efficiency and enhance host protein synthesis (Guoyao et al., 2007), which is consistent with our results. Therefore, the increased nitrogen metabolism in the co-administration of *L. rhamnosus* GG ATCC53103 and *L. plantarum* JL01 in weaned piglets may be mediated by L-Tryptophan, 3-Phosphonooxypyruvate, cis-Aconitate and Carbamoyl phosphate. The increase of these metabolites may be related to the functional genes contained in the significantly increased flora.

## 5. Conclusion

In sum, oral administration of *L. plantarum* JL01 and *L. rhamnosus* GG ATCC53103 to weaned piglets increased apparent nitrogen digestion. We found that dietary supplementation of *L. plantarum* JL01 and *L. rhamnosus* GG ATCC53103 improved the gut microbial diversity of weaned piglets. With the help of functional genes encoded by dominant intestinal bacteria, the production of dominant metabolites L-tryptophan 3-Phosphonooxypyruvate, cis-Aconitate, and Carbamoyl phosphate is promoted, and these metabolites promote the TCA cycle in different ways, which may lead to the increase of AKG, and then play a positive regulatory role in skeletal muscle protein deposition. Moreover, L-tryptophan has a positive regulatory effect on nitrogen metabolism. This finding further highlights the importance of gut microbiota for nitrogen metabolism and demonstrates an effect of *L. plantarum* JL01 and *L. rhamnosus* GG ATCC53103 to promote nitrogen metabolism in weaned piglets.

## Data availability statement

The data presented in the study are deposited in the figshare repository, accession number 10.6084/m9.figshare.23537733.

## Ethics statement

The animal study was reviewed and approved by the Ethics Committee of Jilin Agricultural University.

## Author contributions

FH, XJ, TG, SS, and HS designed. FH wrote this manuscript. CW, JH, LZ, YZ, NB, and LP were involved in modifying the manuscript. HS supervised the writing of this manuscript. All authors read and approved the final version of the manuscript.
